# A machine learning approach to personalized predictors of dyslipidemia: a cohort study

**DOI:** 10.3389/fpubh.2023.1213926

**Published:** 2023-09-20

**Authors:** Guadalupe Gutiérrez-Esparza, Tomas Pulido, Mireya Martínez-García, Tania Ramírez-delReal, Lucero E. Groves-Miralrio, Manlio F. Márquez-Murillo, Luis M. Amezcua-Guerra, Gilberto Vargas-Alarcón, Enrique Hernández-Lemus

**Affiliations:** ^1^Researcher for Mexico CONAHCYT, National Council of Humanities Sciences, and Technologies, Mexico City, Mexico; ^2^Clinical Research, National Institute of Cardiology “Ignacio Chávez”, Mexico City, Mexico; ^3^Department of Immunology, National Institute of Cardiology “Ignacio Chávez”, Mexico City, Mexico; ^4^Center for Research in Geospatial Information Sciences, Aguascalientes, Mexico; ^5^Department of Electrocardiology, National Institute of Cardiology “Ignacio Chávez”, Mexico City, Mexico; ^6^Department of Molecular Biology and Endocrinology, National Institute of Cardiology “Ignacio Chávez”, Mexico City, Mexico; ^7^Computational Genomics Division, National Institute of Genomic Medicine, Mexico City, Mexico; ^8^Center for Complexity Sciences, Universidad Nacional Autónoma de México, Mexico City, Mexico

**Keywords:** hypertriglyceridemia, hypercholesterolemia, hypoalphalipoproteinemia, mixed hyperlipidemias, feature selection, machine learning, *Tlalpan 2020* cohort, Mexico City

## Abstract

**Introduction:**

Mexico ranks second in the global prevalence of obesity in the adult population, which increases the probability of developing dyslipidemia. Dyslipidemia is closely related to cardiovascular diseases, which are the leading cause of death in the country. Therefore, developing tools that facilitate the prediction of dyslipidemias is essential for prevention and early treatment.

**Methods:**

In this study, we utilized a dataset from a Mexico City cohort consisting of 2,621 participants, men and women aged between 20 and 50 years, with and without some type of dyslipidemia. Our primary objective was to identify potential factors associated with different types of dyslipidemia in both men and women. Machine learning algorithms were employed to achieve this goal. To facilitate feature selection, we applied the Variable Importance Measures (VIM) of Random Forest (RF), XGBoost, and Gradient Boosting Machine (GBM). Additionally, to address class imbalance, we employed Synthetic Minority Over-sampling Technique (SMOTE) for dataset resampling. The dataset encompassed anthropometric measurements, biochemical tests, dietary intake, family health history, and other health parameters, including smoking habits, alcohol consumption, quality of sleep, and physical activity.

**Results:**

Our results revealed that the VIM algorithm of RF yielded the most optimal subset of attributes, closely followed by GBM, achieving a balanced accuracy of up to 80%. The selection of the best subset of attributes was based on the comparative performance of classifiers, evaluated through balanced accuracy, sensitivity, and specificity metrics.

**Discussion:**

The top five features contributing to an increased risk of various types of dyslipidemia were identified through the machine learning technique. These features include body mass index, elevated uric acid levels, age, sleep disorders, and anxiety. The findings of this study shed light on significant factors that play a role in dyslipidemia development, aiding in the early identification, prevention, and treatment of this condition.

## 1. Introduction

Dyslipidemia is a metabolic alteration characterized by elevated levels of cholesterol, triglycerides (TGs), and Low-Density Lipoprotein Cholesterol (LDL), as well as a decrease in High-Density Lipoprotein Cholesterol (HDL) levels. Worldwide, dyslipidemia presents as an exponential health problem with severe consequences and is considered one of the main risk factors for ischemic heart disease, cardiovascular disease, stroke, coronary heart disease, and type 2 diabetes mellitus (T2DM), which is the principal cause of death in adults in Mexico (1, 2). Pirillo et al. (3) have pointed out that ischemic heart disease reached a total of 3.78 million deaths in 2019, with high plasma LDL being the principal cause. These authors also reported between 0.61 and 2.73 million deaths due to ischemic stroke, a strongly associated condition. Similarly, there is a high variation in the number of deaths between countries, presumably due to regional differences and types of dyslipidemia. According to the same authors Pirillo et al. (3), low plasma HDL levels have been the most common type of dyslipidemia in Latin America since 2005, followed by hypertriglyceridemia and high plasma LDL levels.

According to the *National Cholesterol Education Program Adult Treatment Panel III (ATP III)* criteria, the classification of lipid profile dyslipidemias includes four types (see Table 1). Hypertriglyceridemia is a common lipid abnormality characterized by elevated triglyceride (TG) levels, often affecting individuals with visceral obesity, metabolic syndrome, and type 2 diabetes mellitus (T2DM) (4, 5). On the other hand, hypercholesterolemia is associated with high levels of LDL or CHOL and may also be present in individuals with a genetic disorder leading to elevated cholesterol levels (6). Hypoalphalipoproteinemia is frequently observed in people with coronary artery disease and is characterized by low levels of plasma high-density lipoproteins (HDL) (7). Finally, mixed hyperlipidemia, a genetic disorder involving higher cholesterol and triglyceride levels, contributes to the development of coronary artery disease.

**Table 1:** Criteria for lipid profile dyslipidemias used in this study.

**Dyslipidemia type**	**CHOL (mg/dL)**	**HDL (mg/dL)**	**TGs**
Hypertriglyceridemia	<200		>150
Hypercholesterolemia	>200		<150
Hypoalphalipoproteinemia		<40	>150
Mixed hyperlipidemias	>200		>150

In general terms, there are potential risk factors such as increased body mass index (BMI), an excessive dietary intake of saturated fat, and a sedentary lifestyle that contribute to developing a given type of dyslipidemia, a highly complex and heterogeneous set of conditions. This fact complicates the prognosis and diagnostics. In this regard, the widespread use of machine learning (ML) has allowed the application of computational intelligence tools as diagnostic tools for medical issues based on data acquired from analyzed patients. Therefore, such ML models (trained by medical guidance) have been successful in helping doctors to determine medical conditions with improved accuracy in a timely manner (8).

A study proposed by Cui et al. (9) uses ML to predict the risk of dyslipidemia in steelworkers by studying a set of standardized outcomes. They acquired the data by surveying anthropometric data, habits, personal status, and working details. Finally, they apply a Recurrent Neural Network (RNN) and Long Short-Term Memory (LSTM) algorithm, showing excellent performance in predicting dyslipidemia in steel and iron industry employees.

Machine learning has emerged as a valuable tool in predicting dyslipidemia and related conditions based on patient data. For instance, Cui et al. (9) used a recurrent neural network and LSTM algorithm to predict dyslipidemia in steelworkers, achieving excellent accuracy. Lee et al. (10) correlated facial characteristics with hypertriglyceridemia using Naive Bayes classifiers, while Pina et al. (11) showed that a neural network outperformed the Dutch lipid score in predicting dyslipidemia in specialized lipid clinics.

Hatmal et al. (12) used ten ML techniques to predict dyslipidemia with an accuracy of 0.75, considering CD36 protein levels, lipid profile, blood sugar, gender, and age. Similarly, Kim et al. (13) classified and predicted overweight/obesity, dyslipidemia, hypertension, and T2DM using a deep neural network model based on nutritional intake data from Korean citizens. For each disease risk, the accuracies achieved were 0.62496, 0.58654, 0.79958, and 0.80896, respectively.

Dyslipidemia is a complex and heterogeneous condition with potential risk factors such as increased BMI, excessive dietary intake of saturated fat, and a sedentary lifestyle. In this context, machine learning models trained on medical data have shown promising results in improving the diagnosis and prognosis of this condition.

However, recent research indicates that the impact of dyslipidemia on cardiovascular health can vary between men and women due to hormonal, genetic, and lifestyle differences. By analyzing gender-specific differences in dyslipidemia, we can identify unique risk profiles, treatment responses, and underlying mechanisms that may contribute to cardiovascular outcomes. Tailoring interventions based on gender-specific dyslipidemia patterns can lead to more targeted and effective therapies, ultimately improving cardiovascular health for both men and women. This approach also highlights the importance of recognizing and addressing gender-related disparities in dyslipidemia management to optimize patient outcomes and reduce the burden of cardiovascular diseases.

Historically, clinical trials are predominantly done in men, excluding women, even in studies with cells and mice (only male). A review studies the significant causes of diseases by bias in sex and gender. The authors express the influence of differences between sex and gender in genetics, implying affection in diagnosing and treating illnesses (14).

In this context, the present work provides a machine-learning approach to characterize the particularities of men and women with a given type of dyslipidemia (hypertriglyceridemia, hypercholesterolemia, hypoalphalipoproteinemia, as well as mixed hyperlipidemias), identifying the association with clinical factors, biochemical screening, family health history, dietary information, and additional risk factors in order to provide features that can be monitored by health authorities to decrease the risk of long-term complications caused by lipid abnormalities in the study population. While dyslipidemia is a significant risk factor for serious diseases, we acknowledge that our analysis does not incorporate a specific time frame within which an individual might develop the disease. Instead, our study aims to elucidate the underlying risk factors associated with dyslipidemia, providing valuable insights into its etiology and contributing factors. The criteria used in this study to classify dyslipidemia types are shown in Table 1.

## 2. Materials and methods

### 2.1. Data

The present study investigates the cross-sectional association between various factors and cardiovascular health outcomes utilizing data collected from the baseline assessment of the *Tlalpan 2020* cohort (15), a longitudinal research project conducted by the National Institute of Cardiology (Instituto Nacional de Cardiolog-a-Ignacio Chvez) in Mexico City.

The dataset used in this study consists of 2,621 participant records and 137 variables related to anthropometric measurements, clinical parameters, biochemical tests, family health history, physical activity, sleep disorders, smoking habits, alcohol consumption, psychological stress levels, and dietary information. The study identified four types of lipid disorders: 696 cases of hypertriglyceridemia (HTG), 402 cases of hypercholesterolemia (HPLC), 608 cases of hypoalphalipoproteinemia (HPLF), and 548 cases of mixed hyperlipidemia (MIX). Regarding data collection, it was carried out as follows:

- The anthropometric measurements, such as weight, height, and waist circumference (WC), were measured following the *International Society for the Advancement of Kinanthropometry* (16); the clinical parameters systolic (SBP) and diastolic blood pressure (DBP) were calculated considering three measures of each one, with a duration of the 3-min gap.
- In the case of the biochemical tests, the blood samples: fasting plasma glucose (FPG), TGs, HDL, LDL, CHOL, uric acid (URIC), and atherogenic index of plasma (AIP) were taken after 12 h of overnight fasting.
- The variables of family health history considered diseases the mother and father suffered, such as diabetes, obesity, hypertension, dyslipidemia, and heart attack.
- The physical activity was classified based on *International Physical Activity Questionnaire* (17) by METs (metabolic equivalents)-minutes/week into three categories low, moderate, and high.
- We used the *Medical Outcomes Study-Sleep 12-item scale* to determine sleep disorders (18, 19).
- Alcohol consumption was estimated by considering if the participant is a current drinker, the frequency, and the number of cups or beers consumed.
- To classify smoking practice, we consider if the participant is a current smoker, an ex-smoker, or if he/she has never smoked. Supplementary Tables 1–3—presents the variables mentioned in this section.
- Regarding dietary information we applied a software tool called *Evaluation of Nutritional Habits and Nutrient Consumption System* (20). This system analyzes the meals consumed by the participant during a day in the last year and calculates the amount of nutrients consumed. The variables corresponding to the *Evaluation of Nutritional Habits and Nutrient Consumption System* are shown in Supplementary Tables 1, 2.

### 2.2. Methods

**Figure 1:**
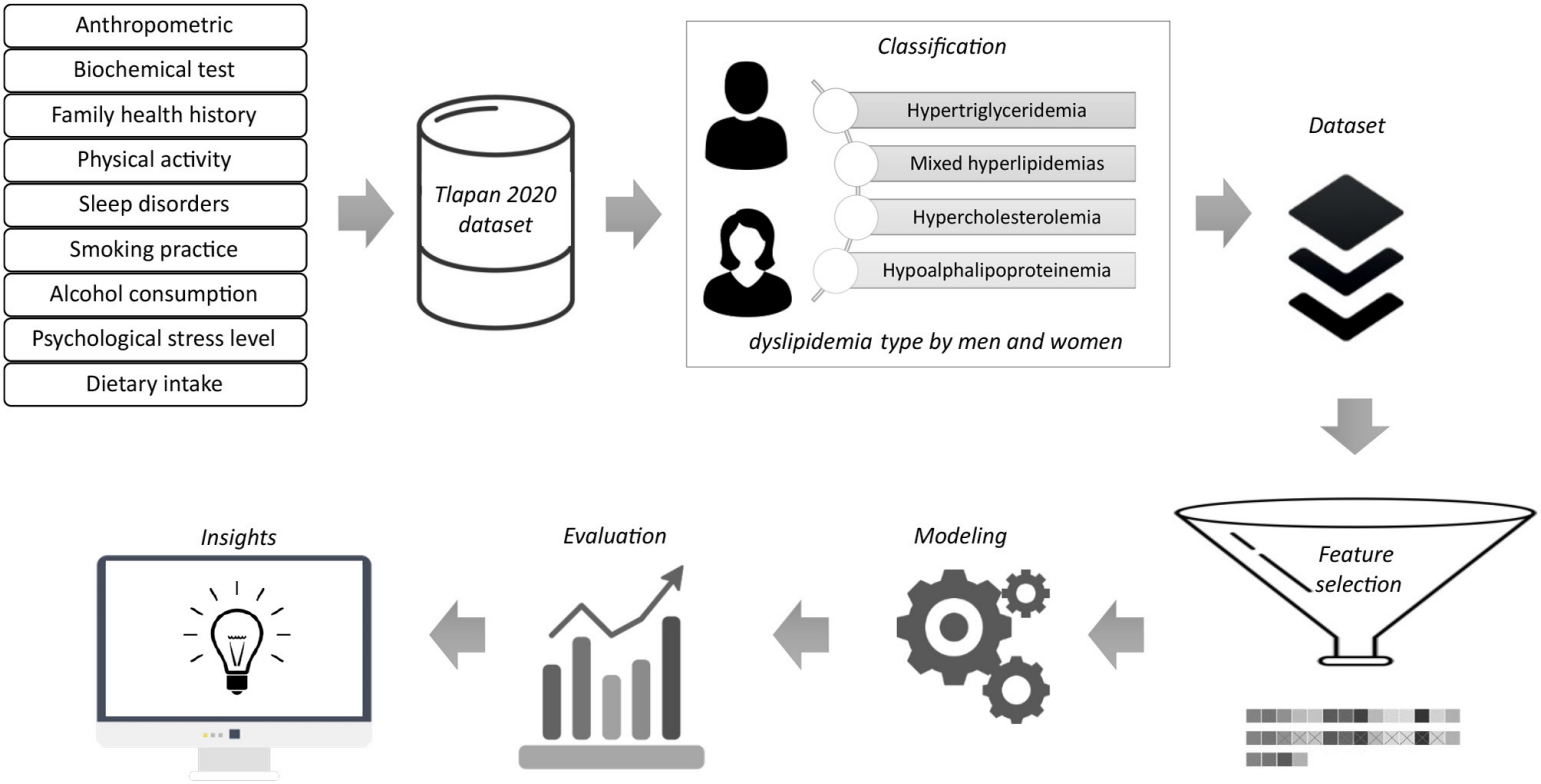
Prediction model.

This work utilized several statistical and data analytics methods. Figure 1 presents the general workflow of the model and describes the methodology used to classify participants with a given type of dyslipidemia and identify the risk factors. Dyslipidemia types were classified according to the ATP III criteria. The dataset was divided into two-thirds for training and the rest for testing. We must note that we applied the SMOTE technique to balance the class distribution in the training dataset.

To find the best subset of variables contributing to improving model performance, we used four methods for feature selection: VIM of RF, XGBoost, RPART, and SHAP. For this study, we applied RF to predict the type of dyslipidemia due to its high performance in diagnosing or predicting dyslipidemia and related diseases (21–23). We developed and evaluated RF performance by running 30 executions using different seeds for each one. To measure the effectiveness of the model, we utilized sensitivity (SENS), specificity (SPC), and balanced accuracy (B.ACC), metrics that have been used for imbalanced data learning assessment. Finally, we obtained the best-performing predictive model.

**Figure 2:**
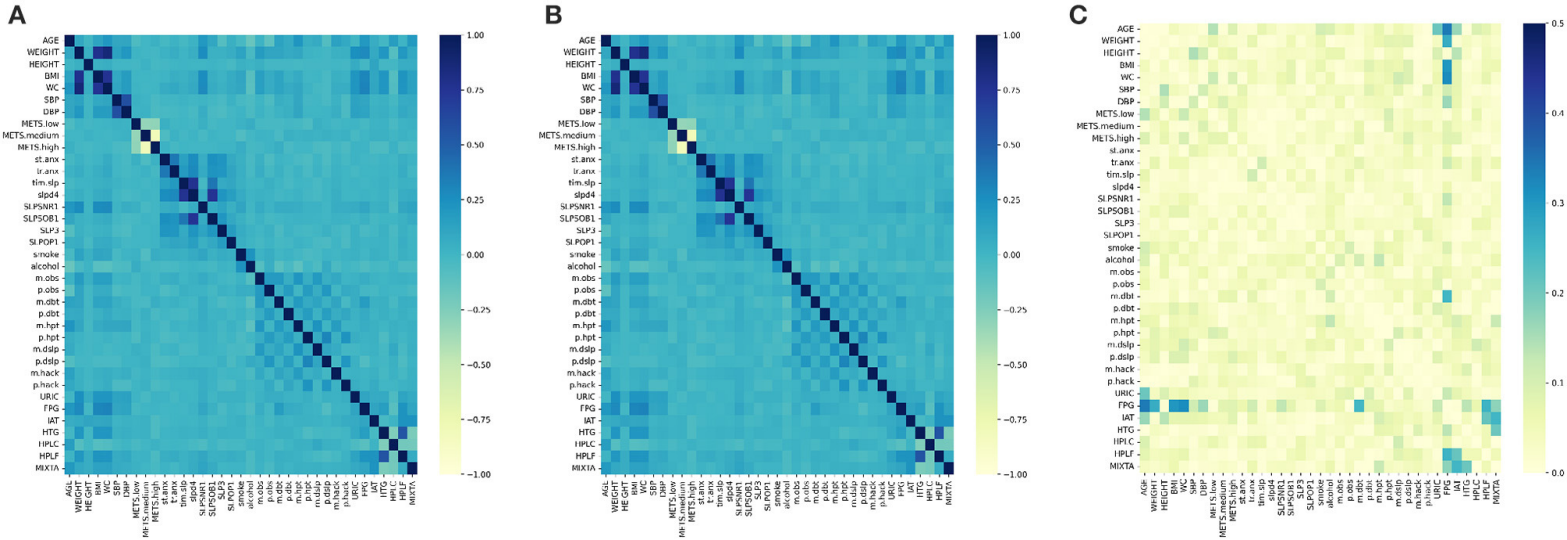
Correlation matrix. **(A)** Women. **(B)** Men. **(C)** Substraction.

The dataset is divided by individuals distinguished by sex (male or female). To justify this division, we perform the correlation matrix with the characteristic variables in addition to the classifications. Figure 2A shows the correlation matrix for women, and Figure 2B shows it for men. The color variation for the correlation is not evident, which is why the subtraction of both is obtained; the result is shown in Figure 2C, where it is evident that there are characteristics that are more related to one gender than to another, in addition to the importance of the difference in the classification of the diagnosis.

#### 2.2.1. Random forest

Random Forest, developed by Breiman et al. (24), is an ensemble machine learning algorithm composed of multiple tree-based estimators for solving classification and regression problems. To reduce over-fitting and improve predictions, this algorithm builds multiple tree-based estimators from training data samples using the Gini index. The Gini index measures the purity of the nodes and can be computed using the following equation:


(1) $$\begin{array}{l} G=\overset{c}{\underset{i=1}{\sum }}p\left(i\right)*\left[1-p\left(i\right)\right] \end{array}$$

where *c* is the number of classes and *p*(*i*) is the proportion of samples that belong to class *c*.

In addition, this algorithm can be used for feature selection by calculating the importance score of variables using the permutation feature importance method.

#### 2.2.2. XGBoost

Extreme Gradient Boosting (XGBoost), presented by Chen and Guestrin (25), is a high-performance ensemble machine learning algorithm that calculates the variable importance by providing a score for each feature.

#### 2.2.3. GBM

GBM is an ensemble model introduced by Friedman et al. (26) that follows the principle of gradient boosting. It consists of a set of individual decision trees, called weak learners, that are trained sequentially to minimize the loss function of the simple models. This model can be computed using the following equation:


(2) $$\begin{array}{l} F\left(x_{i}\right)=\overset{M}{\underset{m=1}{\sum }}vh_{m}\left(x_{i}\right) \end{array}$$

where *y*_*i*_ and *x*_*i*_ are weak learners, with *i*∈(1, ..., *n*) and *i*∈ℤ^+^. The constant *v* (shrinkage factor) is used to control the learning rate, and *h*_*m*_(*x*_*i*_) comes from a decision tree. GBM tries to fit *h*_*m*_(*x*) by minimizing the loss function:


(3) $$\begin{array}{l} \overset{n}{\underset{i=1}{\sum }}\left(y_{i}-F_{m}\left(x_{i}\right)\right) \end{array}$$

#### 2.2.4. Performance measures

To evaluate the performance of models and the different subsets of features, we used the following performance metrics: balanced accuracy (B.ACC), sensitivity (SENS), and specificity (SPC)


(4) $$\begin{array}{l} SENS=\frac{TP}{TP+FN} \end{array}$$

(5) $$\begin{array}{l} SPC=\frac{TN}{FP+TN} \end{array}$$

(6) $$\begin{array}{l} B.ACC=\frac{\left(\frac{TP}{\left(TP+FN\right)}\right)+\left(\frac{TN}{FP+TN}\right)}{2} \end{array}$$

Where *P = Positive, N = Negative, TP = True Positive, FN = False Negative, TN = True Negative, and FP = False Positive*, respectively.

## 3. Experimental setup

We used a 32 GB RAM, 3.50 GHz, Intel Xeon® Dell® Workstation to perform all calculations. **R** v. 3.6.1 with RStudio and Python v. 3.10.7 were used as programming languages. Purposely, these resources are readily available for implementation in most hospital informatics settings.

## 4. Results

The problem of abnormal TG levels can develop based on different factors influencing individuals depending on their lifestyle. Moreover, LDL levels tend to be higher in men than in women until menopause. Hence, in this study, we initially separated the data by gender to obtain the most crucial care features according to the type of dyslipidemia. To identify the potential features by gender and type of dyslipidemia, we applied SMOTE as a resampling method due to class imbalance and three machine learning algorithms, namely VIM of RF, XGBoost, and GBM.

Once we obtained the results from the aforementioned algorithms, we considered displaying at least the top ten most important variables (ranked) that influence each type of dyslipidemia. Each result table shows a different subset of features for each gender and type of dyslipidemia by applying VIM of RF, XGBoost, and GBM.

The results obtained for hypertriglyceridemia are presented in Table 2, followed by the results for hypercholesterolemia in Table 3, as well as the essential variables for hypoalphalipoproteinemia, displayed in Table 4, and finally, the results for mixed hyperlipidemias in Table 5. Summarized general data from the total cohort is presented in Supplementary Table 4.

**Table 2:** Features obtained for prediction of hypertriglyceridemia.

**Random forest**				**Extreme gradient boosting**				**Gradient boosting machine**			
**MEN**	**RANK**	**WOMEN**	**RANK**	**MEN**	**RANK**	**WOMEN**	**RANK**	**MEN**	**RANK**	**WOMEN**	**RANK**
SLPOP1	135.9878	BMI	62.7571	WEIGHT	178	URIC	153	WEIGHT	9.04658472	URIC	15.0950
WHBREADSL	69.1448	WC	47.4358	HEIGHT	149	FPG	128	WC	7.76540979	BMI	14.1867
ANIMALFT	58.8152	SLPSNR1	45.3041	WC	145	BMI	126	AGE	5.3379241	WC	12.6502
TR.ANX	57.88	P.HPT	43.3103	AGE	133	SBP	122	HEIGHT	5.26205646	FPG	11.6328
VEGSHORT	54.99	SLPSOB1	42.7748	SLPD4	122	WEIGHT	120	SLPD4	3.43920487	WEIGHT	6.2303
M.OBS	50.4908	URIC	40.5966	SLPSNR1	69	AGE	110	TR.ANX	2.63816295	SLPD4	5.4541
MAMEYSLC	46.891	WEIGHT	38.9927	TR.ANX	56	DBP	96	SLPSNR1	2.54049134	DBP	5.0677
BMIB	46.3552	COLASMD	38.5838	SMOKE	46	WC	96	SLP3	2.32463614	AGE	4.4456
BLCKCOFE	43.3054	FPG	38.0575	SLP3	44	SLPD4	94	FLAVSODA	2.12517177	SBP	4.1410
GREENBNS2	38.4609	WC	37.6175	ALCOHOL	37	SLP3	74	SMOKE	1.63174985	COLASMD	3.0547
MARGARIN	37.257	TR.ANX	36.8778	SOYAOIL	36	HEIGHT	70	OATMEAL1	1.55762621	ANIMALFT	2.2684
OATMEAL	36.9247	CORNCRLS	35.0434	FPG	29	SLPSNR1	47	SLPSOB1	1.55742209	M.OBS	1.9740
ALCOHOL	36.4578	DBP	34.0421	M.HPT	29	SMOKE	33				

**Table 3:** Features obtained for prediction of hypercholesterolemia.

**Random forest**				**Extreme gradient boosting**				**Gradient boosting machine**			
**MEN**	**RANK**	**WOMEN**	**RANK**	**MEN**	**RANK**	**WOMEN**	**RANK**	**MEN**	**RANK**	**WOMEN**	**RANK**
METS.LOW	18.1341	TR.ANX	18.8690	WEIGHT	120	URIC	160	WEIGHT	12.3907	AGE	9.56756769
SMOKE	18.0843	URIC	16.7090	AGE	103	FPG	127	AGE	7.9561	URIC	6.6324207
P.HPT	13.9113	LIVERSTK	16.6255	WC	86	BMI	124	WC	7.3735	SBP	6.53886356
MARGARIN	13.3063	WHBREADSL	14.9842	HEIGHT	68	SBP	119	HEIGHT	5.8700	BMI	5.9425091
TR.ANX	12.8586	TIM.SLP	14.8968	SLPD4	54	AGE	114	SLP3	4.4935	FPG	5.9132817
WC	12.4936	BMIB	13.9930	SLP3	50	DBP	104	SLPD4	3.6670	FLAVSODA	4.21043414
HEIGHT	11.9679	METS.low	12.1474	SLPSNR1	32	WEIGHT	99	SLPSNR1	3.3304	SLPSNR1	3.76874362
WEIGHT	11.4366	SLPOP1	11.4056	TR.ANX	30	SLPD4	73	SOYAOIL	2.1166	WEIGHT	3.59883532
OATMEAL2	11.0669	OATMEAL	11.3356	OATMEAL1	29	WC	65	OATMEAL1	2.1137	WC	3.38394044
HARDLQUR	10.2940	MARGARIN	11.1782	SMOKE	28	HEIGHT	61	ALCOHOL	2.0129	SLPD4	2.94644812
CHOCPWDR	8.9039	SMOKE	10.8057	WHBREADSL	25	SLP3	55	SMOKE	1.9512	HEIGHT	2.51790709
AGE	8.8006	WEIGHT	9.5272	SLPOP1	24	SLPSNR1	48	DIETCOLA	1.9308	DBP	2.49228445
URIC	8.5430	P.OBS	9.8361	SLP0B1	23	TR.ANX	28				

**Table 4:** Features obtained for prediction of hypoalphalipoproteinemia.

**Random forest**				**Extreme Gradient Boosting**				**Gradient Boosting Machine**			
**MEN**	**RANK**	**WOMEN**	**RANK**	**MEN**	**RANK**	**WOMEN**	**RANK**	**MEN**	**RANK**	**WOMEN**	**RANK**
MARGARIN	94.9750	URIC	45.6856	WEIGHT	143	URIC	145	WEIGHT	11.4121	URIC	20.7737
OATMEAL	62.1306	SLPSNR1	44.5059	HEIGHT	122	SBP	126	WC	10.1244	BMI	13.721
ALCOHOL	62.1098	BMI	39.4508	SLPD4	120	FPG	103	AGE	7.1326	FPG	13.348
BMI	47.3970	WC	38.4695	WC	119	DBP	101	HEIGHT	6.8948	SBP	9.7432
FPG	45.2968	FPG	30.7986	AGE	116	BMI	101	SLPD4	5.3522	WEIGHT	4.9799
SOYAOIL	43.7874	SLPSOB1	27.0383	SLP3	74	AGE	86	SLPSNR1	2.6590	WC	3.8663
TR.ANX	43.4292	WEIGHT	26.7650	SLPSNR1	73	WEIGHT	85	SLP3	2.5206	SLPD4	3.3011
HARDLQUR	42.4482	FPG	25.6375	SMOKE	43	SLPD4	70	ALCOHOL	2.2908	SLP3	3.0089
METS.low	41.2810	SBP	25.4510	P.DSLP	42	HEIGHT	70	SMOKE	2.2221	ALCOHOL	2.7355
DIETCOLA	40.1276	DBP	24.0586	TR.ANX	35	SLP3	59	TR.ANX	1.9325	WHBREADSL	2.4858
SLPOP1	38.4485	ALCOHOL	24.0578	URIC	34	WC	58	CRMCHSPOO	1.4264	ST.ANX	2.0462
LIVERSTK	36.6570	WHBREADSL	21.9719	SLPSOB1	30	SMOKE	35	URIC	1.3727	AGE	1.8986
M.OBS	32.6166	TR.ANX	21.3765			SLPSNR1	33				

**Table 5:** Features obtained for prediction of mixed hyperlipidemias.

**Random forest**				**Extreme Gradient Boosting**				**Gradient Boosting Machine**			
**MEN**	**RANK**	**WOMEN**	**RANK**	**MEN**	**RANK**	**WOMEN**	**RANK**	**MEN**	**RANK**	**WOMEN**	**RANK**
OATMEAL1	111.3498	BMI	54.9825	WEIGHT	134	URIC	127	WEIGHT	7.2914	AGE	15.636
OATMEAL	61.6553	AGE	47.9841	WC	119	SBP	123	OLIVEOIL	7.1260	FPG	14.1844
MARGARIN	46.1016	SLPSNR1	45.7455	AGE	116	BMI	121	WC	6.5050	URIC	13.2189
TABLEWIN	40.2253	FPG	40.0908	HEIGHT	110	DBP	117	HEIGHT	6.4946	BMI	9.0322
SAFFLOWR	38.8777	WC	37.5964	SLPD4	95	FPG	102	AGE	5.7541	HEIGHT	7.0715
HARDLQUR	36.1876	SLPSOB1	29.5068	SLP3	75	AGE	94	SLPD4	4.5321	SBP	6.3664
ALCOHOL	34.9600	URIC	28.1884	SLPSNR1	60	WC	87	PLUMS	3.7759	DBP	4.8929
BMI	34.4313	SMOKE	26.6538	SMOKE	36	WEIGHT	84	SLP3	2.7475	SLPD4	4.6317
P.DSLP	33.0949	HEIGHT	26.3499	SLPSOB1	30	HEIGHT	78	SAFFLOWR	2.2864	WC	2.6707
ZAPOTE	37.7836	TR.ANX	25.2881	ALCOHOL	29	SLPD4	71	SLPSNR1	1.8169	FLAVSODA	2.5535
CRMCHSPOO	28.5640	ALCOHOL	25.2773	M.HPT	28	SLP3	53	ALCOHOL	1.5273	METS.low	2.4744
AGE	28.1579	SBP	25.1633	SLPOP1	27	SLPSNR1	35	BUTTER	1.4225	CORNCRLS	2.0466
OLIVEOIL	27.9041	SUGRDRNK	22.7089	TR.ANX	26	TR.ANX	28				

Subsequently, each algorithm generated subsets of variables, which were used to select the best features. To perform this feature selection process, we applied RF, which was optimized by *grid search method* (27) (resulting in varying *mtry* and *ntree* values for each gender and dyslipidemia type). We employed 10-fold cross-validation with ten repeats to evaluate the performance. Following this, we conducted 30 independent executions with different seeds to ensure robustness and approximate a normal distribution. This approach aligns with similar practices observed in relevant studies (28, 29). The evaluation was based on balanced accuracy, serving as the primary criterion for assessment.

To measure the performance of the RF model, the metrics B.ACC, SENS, and SPC were considered; likewise, it was necessary to apply SMOTE due to the unbalanced dataset. Table 6 shows each result of RF by using the different subset of variables obtained by VIM of RF, XGBoost, RPART, and SHAP, for men and women, as well as the respective parameter tuning and standard deviation (SD).

**Table 6:** Results of random forest using different variable subsets.

			**Random forest**			**Extreme gradient boosting**			**Gradient boosting machine**		
**Dyslipidemia**	**Sex**	**Parameters**	**BACC**	**Sensitivity**	**Specificity**	**BACC**	**Sensitivity**	**Specificity**	**BACC**	**Sensitivity**	**Specificity**
HTG	MEN	mtry = 10	77.44%	85.98%	70.91%	**82.77%**	86.34%	79.20%	77.55%	83.00%	72.09%
		ntree = 200	1.7258	2.8453	2.2075	1.2692	2.1674	1.0794	1.4228	2.4354	1.5687
	WOMEN	mtry = 7	**82.50%**	87.57%	77.43%	73.38%	82.64%	64.12%	80.10%	80.67%	79.54%
		ntree = 500	1.0866	1.8336	1.0639	1.5623	1.9396	2.5682	1.5182	2.5473	1.1955
HPLC	MEN	mtry = 6	76.88%	95.18%	66.04%	72.56%	85.61%	59.51%	**83.69%**	87.91%	79.47%
		ntree = 200	2.6915	1.3835	3.8351	1.3663	2.3122	2.1522	1.5232	2.8082	1.2830
	WOMEN	mtry = 9	**79.74%**	87.45%	72.03%	75.94%	86.35%	65.52%	73.12%	79.84%	71.39%
		ntree = 200	1.1626	2.0079	1.6864	1.7560	2.7484	2.6783	2.0683	2.9511	2.6050
HPLF	MEN	mtry = 10	78.18%	86.77%	71.01%	80.09%	83.04%	77.15%	**80.50%**	83.68%	77.32%
		ntree = 300	1.6872	2.2423	2.2685	1.4658	2.3286	1.3990	1.2918	2.1942	1.3077
	WOMEN	mtry = 7	**83.65%**	87.55%	79.75%	72.95%	82.46%	63.45%	74.30%	87.48%	61.13%
		ntree = 800	1.2227	2.2218	1.5631	1.7576	2.2209	2.7013	1.5893	2.6710	2.5128
MIXED	MEN	mtry = 10	**83.71%**	94.58%	72.84%	73.98%	85.66%	63.30%	83.32%	84.64%	82.01%
		ntree = 200	1.1905	1.5817	2.0563	1.7822	2.4196	2.3847	1.5611	3.0704	1.4224
	WOMEN	mtry = 7	**81.70%**	90.85%	72.55%	76.00%	86.51%	65.49%	73.27%	77.54%	70.00%
		ntree = 100	1.3209	2.2095	1.7068	1.4999	2.2765	2.4131	1.9856	2.9964	3.2172

The bolded values correspond to the models with the highest balanced accuracy based on gender and type of dyslipidemia.

In the case of men with hypertriglyceridemia, the subset of features obtained by XGBoost achieved the best RF performance with a B.ACC of 82.77% and SD of 1.26. The top variables of this subset showed the influence of overweight, where the first three variables are related to it and *body mass index* (BMI), followed by *age, sleep disturbance* (SLPD4) and FYI (SLPSNR1), *anxiety as a trait* (TR.ANX), *smoking practice* (SMOKE), *somnolence* (SLP3), *alcohol consumption* (ALCOHOL), *soy oil consumption* (SOYAOIL), *glucose levels* (FPG), and *medical history of the mother with hypertension* (M.HPT).

Moreover, for women, the subset of variables obtained by VIM of RF achieved the best performance with a B.ACC of 82.50 and an SD of 1.08, where the principal variable was *uric acid levels* (URIC) [several studies (30, 31) have found an association between high uric acid and hypertriglyceridemia]. The other variables in this subset include *glucose levels* (FPG), *body mass index* (BMI), *Systolic blood pressure* (SBP), *weight, age, Diastolic blood pressure* (DBP), *Waist circumference* (WAIST), *sleep disturbance* (SLPD4), *somnolence* (SLP3), *height, snoring* (SLPSNR1), and *smoking practice* (SMOKE). All these variables are considered risk factors contributing to the development of hypertriglyceridemia (32, 33).

For hypercholesterolemia, the variables obtained by GBM achieved the best RF performance for men, with a B.ACC of 83.69% and an SD of 1.52. The principal variables found by this model denote a close relation between being overweight as represented by (WEIGHT, WC, AGE, and HEIGHT), *sleep disturbances* (SLPSNR1, SLP3, and SLPSOB1), *anxiety disorders* (TR.ANX), and habits such as *consumption of flavored soda* (FLAVSODA) and *smoking* (SMOKE).

In the case of women with hypercholesterolemia, the best performance was obtained by the subset generated by VIM or RF with a B.ACC of 79.74% and SD of 1.16, where the *anxiety disorders* (TR.ANX) and *uric acid levels* (URIC) were the principal variables, as well as frequently consuming some foods like *chicken liver* (LIVERSTK), *bread* (WHBREADSL), *oatmeal bowl* (OATMEAL), and *margarine* (MARGARIN), likewise, variables related to sleep disorders like the *time to fall asleep* (TIM.SLP) and *sleep short duration* (SLPOP1), followed by *low physical activity* (METS.low), *smoking* and *history of obese parents* (P.OBS).

For men with hypoalphalipoproteinemia, the best subset of variables was presented by GBM with a B.ACC of 80.50% and SD of 1,29, being variables related to *overweight* the best qualified (WEIGHT, WC, and HEIGHT), as well as *age*, followed by indicators of sleep disorders like *sleep disturbance* (SLPD4), *snoring* (SLPSNR1) and *somnolence* (SLP3). Likewise, habits of *alcohol consumption* and *smoking, anxiety disorder, cream cheese consumption* (CRMCHSPOO) and elevated *uric acid levels* (URIC).

Similarly, the VIM of RF was the best subset of variables for women with hypoalphalipoproteinemia, with a B.ACC of 83.65% and SD of 1.22. In this case, the principal variable was elevated *uric acid levels*, followed by *snoring* (SLPSNR1) and variables closely related to *overweight* (BMI, WC, WEIGHT), as well as *glucose levels* (FPG) and *blood pressure levels* (SBP and DBP) denoted their presence as risk factors, finishing with the consumption of *alcohol* and *bread* (WHBREADSL), as well as *anxiety*.

Finally, the subset of variables obtained by VIM of RF got the best performance for men with mixed hyperlipidemia. In this case, the main variables were closely related to food consumption such as *atole without milk* (OATMEAL1), *oatmeal bowl* (OATMEAL), a *teaspoon of margarine* (MARGARIN), a *glass of table wine* (TABLEWIN), *safflower oil* (SAFFLOWR), *rum, brandy or tequila* (HARDLQUR), *zapote* (FREQ025), a *tablespoon of cream cheese* (FREQ005) and *olive oil* (OLIVEOIL), as well as, ALCOHOL, BMI, history of *a parent with dyslipidaemia* (P.DSLP), and *age*.

For women with mixed hyperlipidemia, the variables obtained by VIM of RF with the best-ranked factors were BMI, *age*, and *snoring*, followed by *glucose levels, waist circumference, sleep short duration, uric acid levels, smoking, height, anxiety, alcohol consumption, Systolic Blood Pressure*, and *a glass of flavored sugar water* (SUGRDRNK).

## 5. Discussion

In what follows, we will discuss the present analysis's expected and novel findings to contextualize the potential value of public health interventions.

In order to determine the significance of studying males and females separately, a significance analysis was conducted using the chi-squared test. The results indicated a strong relationship between gender and the prediction of dyslipidemia types and their critical factors.

The significant associations found for SEX in all dyslipidemias type further emphasize the importance of gender as a significant factor influencing dyslipidemia prediction. Therefore, conducting separate analyses for males and females was crucial to gain a comprehensive understanding of the underlying factors associated with dyslipidemia in each gender group. The results of this significance analysis can be seen in the Supplementary Tables 5–8.

In the case of men with hypertriglyceridemia, several known associations arise. That is the case of *overweight* (34–37), *age* (38, 39), and *waist circumference* (40, 41). Additionally, we discovered a set of relatively new yet significant predictors whose relevance and mechanisms concerning hypertriglyceridemia in men are still to be determined, such as *anxiety, tomato sauce consumption*, and *history of hypertension in the mother*. Regarding the association between *anxiety* and hypertriglyceridemia, van Reedt Dortland and collaborators have identified a potential role of tricyclic antidepressant drugs (42). In contrast, other authors have identified an increased risk of hypertriglyceridemia in patients with psychiatric diseases without relation to specific pharmacological treatment (43).

The case of *tomato sauce consumption* presents some contradictory features. At the same time, some authors have described a protective role of processed tomato products to post-prandial oxidation and inflammation (both associated with dyslipidemias) in *healthy weight* subjects (44–46). In contrast, others have related processed foods (including tomato sauce) to hypertriglyceridemia (47, 48).

Since these studies differ in the methods and types of populations under investigation, differences may be explained by such disparate approaches. Hence definite associations need to be further studied with properly defined research methods.

No previous studies have directly linked *maternal hypertension history* to hypertriglyceridemia. Interestingly however, is the fact that there is an unusual prevalence of hypertriglyceridemia in small populations with known risk factors for pregnancy-associated high blood pressure (49–52), though, at this stage, an actual association is still to be further validated in more extensive population studies.

Similarly, in the case of women with hypertriglyceridemia, the best predictors were some known factors such as AIP (a prominent feature by construction) as well as *BMI, age*, and *cola drink consumption*. Other metabolic features appear, such as glucose and uric acid levels and also *raw tomato consumption*. Regarding the role of high fasting glucose levels in the presence of hypertriglyceridemia, reports have long been made, particularly by driving mechanisms of endogenous hypertriglyceridemia (35, 53, 54). The fact that *FPG* is a better predictor for hypertriglyceridemia in women than in men may be related to the effects of hormone (in particular, estrogen) metabolism in lipid and glucose processing biochemical pathways (55–57).

Elevated *uric acid levels* have been previously associated with hypertriglyceridemia, both in extensive cohort studies (31, 52, 58–60), population-based research (61–64), and biochemically-based analyses (65–69). Unlike processed tomato products, whose effects on hypertriglyceridemia are ambiguous (as previously discussed), *raw tomato consumption* has been acknowledged as a *protective factor* (44) against dyslipidemia in general and hypertriglyceridemia, in particular, (70–73).

Regarding men with hypercholesterolemia, some of the main predictors are (unsurprisingly) meat-based products with high lipid contents such as tacos *al pastor* (shepherd style), *carnitas*, and *longaniza* (74–76). There is evidence that consuming fatty meats, such as beef, pork, and lamb, may contribute to the development of hypercholesterolemia.

For instance, one study published in the American Journal of Clinical Nutrition found that a diet high in saturated fat, such as that found in fatty meats, was associated with an increase in LDL cholesterol that can, in turn, contribute to the development of cardiovascular disease (77). Another study published in the American Journal of Epidemiology found that individuals who consumed a diet high in red and processed meats had a higher risk of developing hypercholesterolemia than those who consumed a diet low in these types (78).

Aside from fatty meat products, other predictors are foods such as chocolate powder, cream cheese and anthropometrics such as weight and height (79, 80). Some evidence, for instance, suggests that chocolate consumption may be associated with a modest reduction in cholesterol levels, although the effect may be small and may depend on the type of chocolate and the individual.

Several studies published in the American Journal of Clinical Nutrition and the European Journal of Clinical Nutrition found that cocoa and chocolate intake was associated with a slight reduction in total cholesterol and low-density lipoprotein (LDL) cholesterol and that the effect of chocolate on cholesterol levels may be influenced by the type of chocolate consumed, with some studies suggesting that dark chocolate may have a more significant effect on cholesterol levels than milk chocolate (81–83). In contrast, another study recalls that these effects may come via activating flavonoid metabolism and anti-oxidant pathways (84).

In the case of women with hypercholesterolemia, there are well-known factors such as *age* (85, 86), *pork rind* (87, 88), *mayonnaise consumption* (89, 90), and *BMI* (91, 92). Other less-known predictors emerge from our study. Such is the case of *sleep disturbance*. Abnormal sleep conditions are gradually being recognized as relevant players in metabolic and cardiovascular diseases (93–95). However, it is noteworthy that most studies relating hypercholesterolemia with sleep disturbances center on the possible effects on sleep induced by drugs such as Pravastatin and Lovastatin (96–100).

The main predictors found for alphalipoproteinemia in men were *waist circumference* and *BMI* (101, 102), as well as conditions such as *anxiety* (103, 104), and *consumption of seafood* (105, 106) and *plums* (107). In contrast, in women, selected features were known metabolic state and anthropometric markers such as *AIP* (108), *glucose levels* (109, 110), *BMI* and *waist circumference* (101, 102), also *uric acid levels* (111, 112); consumption of high fat or high caloric foods like *pork meat, flavored soda, Oaxaca cheese*, and *bacon* (113). Interestingly *snoring* while sleeping was also a relevant predictor for alphalipoproteinemia in women. Though a direct association of snoring with female alphalipoproteinemia has not been reported, a population-based study has indeed associated self-reported snoring with dyslipidemia, high total cholesterol, and high low-density lipoprotein cholesterol in obese individuals in rural China (114).

Mixed hyperlipidemias in men were best predicted by: *AIP* (115, 116), *waist circumference, BMI* (117, 118), *age* (119), as well as *dried chile peppers consumption* (DRYCHILES) (120–122), as well as drinking *whole milk* (MILKGLASS) (123, 124), *alcohol* (125–127), *sweet bread* (SWEETBRD) (128, 129), and *orange* (ORANGE) intake (130–132). In the case of women with mixed hyperlipidemias top predictive features were: *BMI* (117, 118), *age* (119), *snoring* (114), *glucose levels* (133, 134), *uric acid levels* (64), *smoking* (133), and *anxiety* (135), but also *alcohol* (125, 136) and *flavored sugar water* (BACONSLC) (137, 138) consumption.

## 6. Conclusions

By focusing on identifying risk factors without a time frame, our study lays the foundation for future investigations that could incorporate temporal aspects for predicting the onset of dyslipidemia or subsequent development of CVD. The findings from our research can serve as a basis for developing predictive models that integrate time-based parameters, enabling more accurate and clinically relevant disease prognosis and management.

In this work, the application of machine learning models in a cohort of Mexico City allowed the identification of subsets of attributes acting as risk factors associated with several types of dyslipidemias. Multi-feature diagnostics, i.e., the diagnosis based on different aspects, is considered essential to support healthcare providers as it allows early detection of patients at the most significant risk of developing a type of dyslipidemia, which supports the development of strategies for prevention, treatment, and prognosis the condition.

The separation by gender allowed the discovery of differences between subsets of risk factors associated with each type of dyslipidemia.

Even when we obtained high-performance models with this particular data and the support of SMOTE, it is possible to note that the best classifiers identified risk factors in men with hypercholesterolemia (with a B.ACC of 83.69%) and women with hypoalphalipoproteinemia (with a B.ACC of 83.65%). Therefore, the exploration of other ML models and the continuous update of the data set may not be ruled out in future work to improve the values of the metrics and predict the development of dyslipidemia types.

## Data availability statement

The original contributions presented in the study are included in the article/Supplementary material, further inquiries can be directed to the corresponding author/s.

## Ethics statement

The studies involving humans were approved by Research Ethics Board for Biomedical Research in Humans by the National Institute of Cardiology Ignacio Chavez-Protocol approved with key 13-802. The studies were conducted in accordance with the local legislation and institutional requirements. Written informed consent for participation was not required from the participants or the participants' legal guardians/next of kin in accordance with the national legislation and institutional requirements.

## Author contributions

GG-E designed computational strategy, implemented computing code and algorithmics, evaluated performance measures, co-supervised the project, and drafted the manuscript. TRP-Z guided the clinical approach and provided feedback to the modeling. TR-d implemented computing code and algorithmics and contributed to drafting the manuscript. MM-G performed clinical, sociomedical, and health policy research contributed to drafting the manuscript. LG-M supported data curation. MFM-M performed a clinical assessment. LMA-G contributed to clinical assessment. LMA-G, TRP-Z, and GV-A reviewed clinical results. EH-L devised the overall study strategy, co-supervised the project, performed the technical assessment, and revised and edited the manuscript. All authors read and approved the submitted version of the manuscript.
