# Supplementary material for: A machine learning approach to personalized predictors of dyslipidemia: a cohort study
Source: Front Public Health. 2023 Sep 20;11:1213926. doi: 10.3389/fpubh.2023.1213926 (PMC10548235; doi:10.3389/fpubh.2023.1213926)
Supplement: Supplementary file 1 [file Data_Sheet_1.PDF]

A Machine Learning Approach to Personalized Predictors of Dyslipidemia: A Cohort Study (Supplementary material)

Guadalupe Gutiérrez-Esparza [1,2,*,†], Tomas R. Pulido-Zamudio [2,†], Mireya Martínez-García [2], Tania Ramírez-delReal [1,3], Lucero E. Groves-Miralrio [2], Manlio F. Márquez-Murillo [2], Luis M. Amezcua Guerra [2], Gilberto Vargas-Alarcón [2] and Enrique Hernández-Lemus [4,5,*]

[1] *Researcher for Mexico CONAHCYT, National Council of Humanities, Sciences, and Technologies, México City, México*
[2] *National Institute of Cardiology 'Ignacio Chávez', México City, México*
[3] *Center for Research in Geospatial Information Sciences, Aguascalientes, México*
[4] *Computational Genomics Division, National Institute of Genomic Medicine, México City, México*
[5] *Center for Complexity Sciences, Universidad Nacional Autónoma de México, México City, México*
† *These two authors are joint co-first authors*

Correspondence*:
Enrique Hernández-Lemus
ehernandez@inmegen.gob.mx

Guadalupe Gutiérrez-Esparza
ggutierreze@conacyt.mx

Table 1. Dataset variables (A)

Abbreviation	Variable Name	Type
AGE	age	Continuous
SEX	sex	Dichotomous
WEIGHT	weight	Continuous
HEIGHT	height	Continuous
BMI	BMI	Continuous
WAIST	waist	Continuous
SBP	systolic blood pressure	Continuous
DBP	diastolic blood pressure	Continuous
METS.low	metabolic equivalents low	Dichotomous
METS.medium	metabolic equivalents medium	Dichotomous
METS.high	metabolic equivalents high	Dichotomous
ST.ANX	state anxiety	Dichotomous
TR.ANX	trait anxiety	Dichotomous
TIM.SLP	time to fall asleep	Range from 1 to 3
SLPD4	sleep disturbance	Continuous
SLPSNR1	snoring	Continuous
SLPSOB1	sleep short (headache)	Continuous
SLP3	somnolence	Continuous
SLPOP1	optimal sleep	Dichotomous
SMOKE	smoking practice	Range from 0 to 2
ALCOHOL	alcohol consumption	Range from 0 to 3
M.OBS	obesity mother	Dichotomous
P.OBS	obesity father	Dichotomous
M.DBT	diabetic mother	Dichotomous
P.DBT	diabetic father	Dichotomous
M.HPT	hypertension mother	Dichotomous
P.HPT	hypertension father	Dichotomous
M.DSLP	dyslipidemia mother	Dichotomous
P.DSLP	dyslipidemia father	Dichotomous
M.HACK	heart attack mother	Dichotomous
P.HACK	heart attack father	Dichotomous
URIC	uric acid	Dichotomous
HDL	high-density lipoprotein	Dichotomous
LDL	low-density lipoprotein	Dichotomous
FPG	fasting plasma glucose	Dichotomous
AIP	atherogenic index of plasma	Dichotomous
CHOL	cholesterol	Dichotomous
TGs	triglycerides	Dichotomous
NA	sodium	Dichotomous
mts.atp3	metabolic syndrome	Dichotomous
HTG	hypertriglyceridemia	Dichotomous
HPLC	hypercholesterolemia	Dichotomous
HPLF	hypoalphalipoproteinemia	Dichotomous
MIX	mixed hyperlipidemias	Dichotomous

tim.slp is 1 = good, 2 = regular, 3 = bad. SLPSNR1 is 1 = 100, 2 = 80, 3 = 60, 4 = 40, 5 = 20, 6 = 0, being the value of 100, the bigger problem. SLPSOB1 is 1 = 100, 2 = 80, 3 = 60, 4 = 40, 5 = 20, 6 = 0, being the value of 100, the bigger problem. SLP3 is 1 = 100, 2 = 80, 3 = 60, 4 = 40, 5 = 20, 6 = 0, being the value of 100, the bigger problem.

Table 2. Dataset variables (B)

Abbreviation	Variable name	Type
MILKGLASS	a glass of whole milk	Range from 0 to 9
CHEESESLC	cottage cheese	Range from 0 to 9
OAXCHEESE	Oaxaca cheese	Range from 0 to 9
MNCHCHESE	manchego cheese	Range from 0 to 9
CRMCHSPOO	a tablespoon of cream cheese	Range from 0 to 9
YOGHURTCS	a cup of yogurt	Range from 0 to 9
MILKICECR	milk ice cream	Range from 0 to 9
BANANA	a banana	Range from 0 to 9
ORANGE	an orange	Range from 0 to 9
ORGJUICE	orange/grapefruit juice	Range from 0 to 9
MELONSLC	melon slice	Range from 0 to 9
APPLESLC	an apple	Range from 0 to 9
WATRMLN	a slice of watermelon	Range from 0 to 9
PINEAPPLE	a slice of pineapple	Range from 0 to 9
PAPAYASLC	a slice of papaya	Range from 0 to 9
PEAR	a pear	Range from 0 to 9
MANGO	a mango	Range from 0 to 9
TANGERINE	a tangerine	Range from 0 to 9
STRAWBRY	a portion of strawberries (+/-10)	Range from 0 to 9
APRICOT	a peach	Range from 0 to 9
GRAPES	a portion of grapes (+/-10-15)	Range from 0 to 9
PRICKLY	Prickly Pear (Cactus Fruit)	Range from 0 to 9
PLUMS	a portion of plums (+/- 6)	Range from 0 to 9
MAMEYSLC	a slice of mamey	Range from 0 to 9
ZAPOTE	sapote	Range from 0 to 9
CHICKEGG	chicken egg	Range from 0 to 9
CHICKEN	piece of chicken	Range from 0 to 9
HAMSLC	one slice of ham	Range from 0 to 9
BEEFPLATE	beef (a plate)	Range from 0 to 9
PORKPLATE	pork meat	Range from 0 to 9
TUNAPORT	can of tuna	Range from 0 to 9
PORKSKN	pork rind	Range from 0 to 9
SAUSAGE	a sausage	Range from 0 to 9
BACONSLC	a bacon slice	Range from 0 to 9
LIVERSTK	chicken liver	Range from 0 to 9
CHORIZO	longaniza	Range from 0 to 9
FISHPLATE	mojarra (a plate)	Range from 0 to 9
SARDINAS	sardines in tomato	Range from 0 to 9
SEAFOOD	half cup of maricos	Range from 0 to 9
CARNITAS	carnitas (a plate)	Range from 0 to 9
BARBACOA	barbecue (a plate)	Range from 0 to 9
TOMATOSAU	tomato sauce	Range from 0 to 9
TOMATO	raw tomato	Range from 0 to 9
POTATO	a potato	Range from 0 to 9
CARROTS	1/2 cup of carrots	Range from 0 to 9
LETTUCE	a leaf of lettuce	Range from 0 to 9
SPINACH	1/2 cup of spinach	Range from 0 to 9
ZUCCHINI	1/2 cup of Zucchini	Range from 0 to 9
NOPAL	1/2 cup of cactus	Range from 0 to 9
VEGSOUP	vegetable cream soup	Range from 0 to 9
AVOCADO	half avocado	Range from 0 to 9
SQUASHFLR	1/2 cup of pumpkin flower	Range from 0 to 9
CAULIFLR	1/2 cup of cauliflower	Range from 0 to 9

Table 3. Dataset variables (C)

Abbreviation	Variable name	Type
GREENBNS	1/2 cup green beans	Range from 0 to 9
HOTSCE	hot sauce (a teaspoon)	Range from 0 to 9
CNDCHILES	canned chili	Range from 0 to 9
DRYCHILES	dried chile peppers	Range from 0 to 9
CORN	earn of corn	Range from 0 to 9
BEANSPLATE	beans (a plate)	Range from 0 to 9
PEAS	1/2 cup of green peas	Range from 0 to 9
GREENBNS2	green beans (a plate)	Range from 0 to 9
DRYBNSPLT	dried beans (a plate)	Range from 0 to 9
LENTILS	lentils or chickpeas (a plate)	Range from 0 to 9
CORNTORT	corn tortilla	Range from 0 to 9
WHEATTORT	flour tortilla	Range from 0 to 9
WHBREADSL	loaf of bread	Range from 0 to 9
WHBREADIT	loaf of wholemeal bread	Range from 0 to 9
BUN	telera bread	Range from 0 to 9
SWEETBRD	sweet bread	Range from 0 to 9
RICEPLATE	bowl of rice	Range from 0 to 9
PASTASOUP	pasta soup	Range from 0 to 9
OATMEAL	oatmeal bowl	Range from 0 to 9
CORNCRLS	box cereal bowl	Range from 0 to 9
CAKESLICE	a slice of cake	Range from 0 to 9
SWTSPREAD	A teaspoon of honey, caramel sauce or condensed milk	Range from 0 to 9
CHOCPWDR	A teaspoon of chocolate powder	Range from 0 to 9
CHOCBAR	a tablet of chocolate	Range from 0 to 9
CHIPSBAG	a bag of chips	Range from 0 to 9
COLASMD	medium cola soda	Range from 0 to 9
FLAVSODA	flavored soda	Range from 0 to 9
DIETCOLA	A diet soda	Range from 0 to 9
SUGRDRNK	A glass of flavored sugar water	Range from 0 to 9
BLCKCOFE	A cup of coffee without sugar	Range from 0 to 9
OATMEAL1	A cup of atole without milk	Range from 0 to 9
OATMEAL2	A cup of atole with milk	Range from 0 to 9
BEER	a beer	Range from 0 to 9
TABLEWIN	a glass of table wine	Range from 0 to 9
HARDLQUR	rum, brandy or tequila	Range from 0 to 9
CORNOIL	corn oil	Range from 0 to 9
SOYAOIL	soy oil	Range from 0 to 9
SUNFLOWR	sunflower oil	Range from 0 to 9
SAFFLOWR	Safflower oil	Range from 0 to 9
OLIVEOIL	olive oil	Range from 0 to 9
MARGARIN	a teaspoon of margarine	Range from 0 to 9
BUTTER	a teaspoon of butter	Range from 0 to 9
CREAM	a teaspoon of cream	Range from 0 to 9
MAYONNAI	a teaspoon of mayonnaise	Range from 0 to 9
VEGSHORT	a teaspoon of vegetable shortening	Range from 0 to 9
ANIMALFT	a teaspoon of animal lard	Range from 0 to 9
PASTRTAC	tacos al pastor	Range from 0 to 9
SOPEQUES	a sope or quesadilla	Range from 0 to 9
POZOLEPL	Pozole	Range from 0 to 9
TAMALE	a tamal	Range from 0 to 9

Table 4. General characteristics between male and female among the four types of dyslipidemias

Characteristics	HTG (696)	HPLC (402)	HPLF (608)	MIXED (548)
**Sex**				
Female	359 (51.58)	299 (74.38)	238 (39.14)	259 (47.26)
Male	337 (48.42)	103 (25.62)	370 (60.86)	289 (52.73)
**Age (Years)**				
Female	41 (32-46)	44 (38-48)	41 (33-46)	41 (33-46)
Male	39 (32-44)	42 (32-47)	40 (33-45)	40 (33-45)
**SES, Very low**				
Female	61 (8.76)	34 (8.46)	44 (7.24)	32 (5.83)
Male	42 (6.03)	11 (2.74)	45 (7.40)	41 (7.48)
**SES, Low**				
Female	139 (19.97)	111 (27.61)	91 (14.97)	101 (18.43)
Male	130 (18.68)	33 (8.21)	136 (22.37)	91 (16.61)
**SES, Medium**				
Female	82 (11.78)	68 (16.92)	54 (8.88)	81 (14.78)
Male	84 (12.07)	26 (6.47)	97 (15.95)	81 (14.78)
**SES, High**				
Female	67 (9.62)	84 (20.90)	42 (6.91)	42 (7.66)
Male	71 (10.20)	33 (8.21)	82 (13.49)	73 (13.32)
**Former smoker (Yes)**				
Female	61 (8.76)	46 (11.44)	40 (6.58)	49 (8.94)
Male	62 (8.91)	26 (6.47)	60 (9.87)	47 (8.58)
**Currently smoke (Yes)**				
Female	144 (20.69)	110 (27.36)	100 (16.45)	97 (17.70)
Male	200 (28.74)	46 (11.44)	219 (36.02)	170 (31.2)
**Alcohol consumption (Yes)**				
Female	218 (31.32)	184 (45.77)	144 (23.68)	155 (28.28)
Male	247 (35.49)	80 (19.90)	272 (44.73)	231 (42.15)
**SBP (mmHg)**				
Female	108 (100-116)	105 (99-113)	109 (101-119)	109 (101-119)
Male	113 (106-121)	110 (101-117)	114 (107-121)	114 (107-121)
**DBP (mmHg)**				
Female	73 (68-80)	70 (65-77)	74 (69-79)	74 (69-79)
Male	77 (71-83)	75 (70-80)	79 (71-84)	79 (71-84)
**Waist size (cm)**				
Female	93 (85-100)	86 (80-93)	93.50 (87-102)	93.50 (87-102)
Male	97.50 (91-106)	93 (86-98)	99.50 (93-107)	99.50 (93-107)
**Weight (Kg)**				
Female	70.40 (63-80)	62.80 (57.50-71)	73.10 (63.20-81.80)	73.10 (63.2-81.80)
Male	82.50 (75.20-92)	76 (68.70-85.20)	83.80 (76.30-92.90)	83.80 (76.30-92.90)
**BMI (Kg/m^2))**				
Female	28.75 (25.68-32.26)	25.79 (23.20-29.03)	29.47 (26.33-32.46)	29.47 (26.33-32.46)
Male	82.50 (75.20-92)	76 (68.70-85.20)	83.80 (76.30-92.90)	81 (72.90-89)

The values are expressed in n (%) or med (IQR 25-75) accordingly. *SES*, socioeconomic status.
HTG, hypertriglyceridemia; *HPLC*, hypercholesterolemia; *HPLF*, hypoalphalipoproteinemia; *MIXED*, mixed hyperlipidemias.

Table 5. Results of significance analysis for Hypertriglyceridemia using the chi-Square test

VARIABLE	CHISQUARED	PVALUE
BMI	121.728512	3.69E-27
SEX	35.7592789	2.23E-09
URIC	18.4307479	1.76E-05
IAT	34.6873496	3.05E-05
DBP	14.269506	0.00015841
SMOKE	12.5472538	0.00188538
M.DBT	4.63429845	0.03133896
METS.HIGH	4.00616458	0.04533417
P.DBT	3.81099459	0.05091721
SBP	3.44198838	0.06355916
METS.LOW	2.70540922	0.10000842
TIM.SLP	4.36375068	0.11282974
TR.ANX	6.74655172	0.1499035
P.HPT	1.57791056	0.20906192
SLPOP1	1.50254573	0.22028008
M.HPT	1.24791908	0.26395029
METS.MEDIUM	0.70983802	0.39949759
M.OBS	0.70266576	0.40188941
P.DSLP	0.61243354	0.43387301
ST.ANX	3.57168068	0.46706317
P.HACK	0.28954638	0.59051137
M.HACK	0.04300306	0.83571951
M.DSLP	0.0337458	0.85424858
ALCOHOL	0.70474825	0.87208655
P.OBS	8.49E-29	1

Table 6. Results of significance analysis for Hypercholesterolemia using the chi-Square test

VARIABLE	CHISQUARED	PVALUE
SEX	34.4584966	4.35E-09
FPG	39.3558214	4.22E-06
BMI	22.9261009	1.05E-05
IAT	32.6875589	7.01E-05
M.DSLP	11.5708444	0.00066994
WC	10.5151586	0.00118399
URIC	10.474551	0.0012103
M.HPT	5.10999471	0.02378839
SMOKE	7.18757975	0.02749393
P.HPT	4.01236867	0.04516765
M.HACK	2.7274307	0.09863785
SBP	2.35715003	0.12470984
DBP	1.99955332	0.15734557
METS.HIGH	1.22291828	0.2687887
P.DSLP	0.9428261	0.3315521
METS.LOW	0.78774161	0.37478389
SLPOP1	0.60369551	0.43717149
M.OBS	0.41649325	0.51869205
TIM.SLP	1.27533445	0.52852392
P.OBS	0.2073611	0.64884365
METS.MEDIUM	0.20235501	0.65282663
P.DBT	0.16658969	0.68316062
M.DBT	0.11616832	0.73322799
ALCOHOL	1.10337875	0.7762585
TR.ANX	1.29367002	0.86244859
ST.ANX	1.11427225	0.89200119
P.HACK	6.09E-30	1

Table 7. Results of significance analysis for Hypoalphalipoproteinemia using the chi-Square test

VARIABLE	CHISQUARED	PVALUE
IAT	230.066549	2.87E-45
BMI	171.691527	5.22E-38
SEX	159.699509	1.32E-36
FPG	162.307065	5.26E-31
URIC	75.8344776	3.08E-18
DBP	51.2738742	8.03E-13
SMOKE	24.1885787	5.59E-06
SBP	18.8848575	1.39E-05
M.DBT	8.21340183	0.00415821
SLPOP1	6.45199649	0.01108277
P.HACK	5.61213246	0.01783654
METS.LOW	4.20003299	0.04042319
P.DBT	3.71916769	0.05379118
METS.HIGH	2.90480325	0.08831603
TR.ANX	7.98501259	0.09212875
M.OBS	1.92482049	0.16532623
TIM.SLP	3.47171907	0.17624864
ALCOHOL	2.60389677	0.4568068
P.DSLP	0.54782209	0.45920896
ST.ANX	3.54817827	0.47059065
M.HPT	0.14266723	0.70564369
M.DSLP	0.13983204	0.70844804
P.HPT	0.07283278	0.78725605
METS.MEDIUM	0.07277703	0.78733552
P.OBS	0.04083798	0.83985099
M.HACK	0.00182238	0.9659492

Table 8. Results of significance snalysis for Mixed hyperlipidemias using the chi-Square test

VARIABLE	CHISQUARED	PVALUE
FPG	295.930074	3.05E-29
IAT	122.165038	1.18E-22
SEX	55.267278	1.05E-13
BMI	58.4504945	2.03E-13
URIC	38.0613581	6.86E-10
DBP	19.3446348	1.09E-05
SBP	18.9837078	1.32E-05
SMOKE	6.82593101	0.03294336
METS.LOW	3.80162327	0.05120292
P.DSLP	3.7332325	0.05334007
M.DBT	3.01855573	0.08231675
P.HACK	2.67426964	0.1019821
METS.HIGH	2.47269885	0.11583887
ALCOHOL	4.55123467	0.20776555
TIM.SLP	2.89954477	0.23462369
M.DSLP	1.30667242	0.25299802
M.HPT	0.87667245	0.34911469
TR.ANX	3.1744033	0.52907515
SLPOP1	0.37949548	0.53787337
M.OBS	0.25048115	0.61673649
P.HPT	0.16179548	0.68750881
P.DBT	0.1170638	0.73224111
P.OBS	0.05664604	0.81187777
M.HACK	0.04712505	0.82814363
METS.MEDIUM	0.04147009	0.83863323
